# Starch-Chitosan Polyplexes: A Versatile Carrier System for Anti-Infectives and Gene Delivery

**DOI:** 10.3390/polym10030252

**Published:** 2018-03-01

**Authors:** Hanzey Yasar, Duy-Khiet Ho, Chiara De Rossi, Jennifer Herrmann, Sarah Gordon, Brigitta Loretz, Claus-Michael Lehr

**Affiliations:** 1 Helmholtz Institute for Pharmaceutical Research Saarland (HIPS), Helmholtz Center for Infection Research (HZI), Saarland University, D-66123 Saarbrücken, Germany; Hanzey.Yasar@helmholtz-hzi.de (H.Y.); DuyKhiet.Ho@helmholtz-hzi.de (D.-K.H.); Chiara.DeRossi@helmholtz-hzi.de (C.D.R.); jennifer.herrmann@helmholtz-hzi.de (J.H.); S.C.Gordon@ljmu.ac.uk (S.G.); Claus-Michael.Lehr@helmholtz-hzi.de (C.-M.L.); 2 Department of Pharmacy, Saarland University, D-66123 Saarbrücken, Germany

**Keywords:** polymeric nanoparticles, renewable polysaccharides, anionic starch, cationic anti-infectives, transfection

## Abstract

Despite the enormous potential of nanomedicine, the search for materials from renewable resources that balance bio-medical requirements and engineering aspects is still challenging. This study proposes an easy method to make nanoparticles composed of oxidized starch and chitosan, both isolated from natural biopolymers. The careful adjustment of C/N ratio, polymer concentration and molecular weight allowed for tuning of particle characteristics. The system’s carrier capability was assessed both for anti-infectives and for nucleic acid. Higher starch content polyplexes were found to be suitable for high encapsulation efficiency of cationic anti-infectives and preserving their bactericidal function. A cationic carrier was obtained by coating the anionic polyplex with chitosan. Coating allowed for a minimal amount of cationic polymer to be employed and facilitated plasmid DNA loading both within the particle core and on the surface. Transfection studies showed encouraging result, approximately 5% of A549 cells with reporter gene expression. In summary, starch-chitosan complexes are suitable carriers with promising perspectives for pharmaceutical use.

## 1. Introduction

Nanoparticulate carrier systems represent a well established platform for vaccination and treatment of severe diseases, such as infection and cancer, by protecting active agents, preventing burst release kinetics, providing the potential to enhance crossing of biological barriers and improving local drug delivery [1,2,3,4]. However, the selection of materials or excipients for nanomedical applications remains challenging due to strict requirements of the field. Such materials should be biocompatible and biodegradable, safe and at the same time provide good drug loading capacity as well as a potential to carry diverse bioactive agents [3]. Moreover, for large scale production, the used materials should be environmentally friendly, and able to be manufactured by facile processes. In recent years, a variety of polymeric materials derived from natural biopolymers have been synthesized and investigated to formulate vehicles to deliver bioactive molecules. These molecules have been embedded inside the polymeric matrix or adsorbed onto the colloidal surface [5] by either physical interaction (e.g., electrostatic complexation) or chemical modification. Nevertheless, the number of biodegradable and biocompatible polymers which are further compatible with water (as a solvent suitable for pharmaceutical use) and can form nanoparticles with a high and versatile active agent encapsulation capacity are still limited. Hence, the production of excipients for nanomedicine with a balance between pharmaceutical requirements and engineering aspects as well as a tunable potential for drug delivery has gained considerable attention. In particular, natural and modified polysaccharides such as chitosan, alginate, starch and dextrin, and their synthetic derivatives, have been considered as efficient candidates for drug carrier systems [1,6,7]. However, achieving a consistent and robust production of polysaccharide nanoparticles is challenging due to the heterogeneous physicochemical properties of natural and synthetic polymers. In addition, depending on the actives to be delivered and the route of administration, different protocols are needed [8] to prepare polysaccharide-based polymeric nanoparticles [9,10]. Thus, the chosen polymers need to be appropriately tailored, chemically modified and optimized to qualify for targeted applications [1].

Among natural polysaccharides, starch and chitosan have many promising properties. Starch is a biocompatible and biodegradable polysaccharide, which is degraded by α-amylase, and available at relatively low cost. It has been widely used in tablets and capsules, e.g., as a binder or diluent [11]. Slightly modified derivatives of starch with fractional molecular weights have previously been studied as a platform to formulate homogenous carrier systems for gene delivery [12]. Other researchers have also studied starch-based particulate systems for drug delivery [13,14,15]. Chitosan is similarly biodegradable and biocompatible, and has been investigated and widely used in pharmaceutical research for drug [16,17], protein [18] and nucleic acid delivery, and for vaccination purposes [19,20,21]. It has also been used as a biomedical material for artificial skin and wound healing bandages [22] as a biodegradable polysaccharide [23]. Moreover, chitosan has good biocompatibility as tested in humans [24]. Yamada et al. [12] has reported the preparation of anionic starch derivatives by mild chemical modification, and the separation of different molecular weights by a fractional cut-off protocol, which was later aimed for transfection study. The research showed promising perspectives of starch derivatives as drug carrier system. However, the charge mediated complexation of fractional starch derivatives was not fully explored in that study; the carrier capacity of such system thus remains to be investigated.

**Scheme 1:**
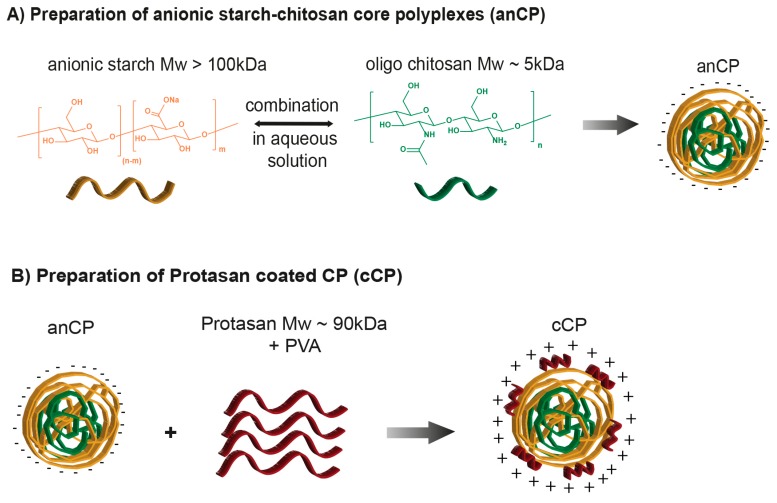
Illustration of drug-free (plain) starch-chitosan polyplex-preparation.

In light of these advantages, the aim of this work was to produce versatile and flexible nanocarriers using both starch and chitosan, with a facile and organic solvent-free preparation method combining the advantages of these two polymers into a carrier system. The investigated systems were composed of starch derivatives of molecular weight (*M*_w_) >100 kDa or with *M*_w_ range of 30–100 kDa, and oligochitosan *M*_w_ 5 kDa or Protasan *M*_w_ 90 kDa as chitosan derivatives. A wide range of molecular weights was used to achieve complex stability. We also explored the design space of the system to obtain particles with high colloidal stability as well as tunable surface charge and size. Thus, the varied production parameters of starch-chitosan polyplexes (Scheme 1A) were: (i) molar ratio of carboxylate and amine functional groups (C/N ratio) of starch and chitosan, respectively; (ii) polymer concentration; and (iii) counter polymer type. The loading capacity and versatility of these simple carriers was then investigated using tobramycin and colistin as clinically relevant models of small molecule and peptide anti-infectives respectively [25,26], as well as nucleic acids (plasmid DNA). Furthermore, to improve encapsulation capacity, we coated the starch-chitosan polyplexes with an additional chitosan (Protasan) layer (Scheme 1B), and explored the loading capacity of the resulting nanoparticles. Coating the polyplexes enabled drug loading on the surface of particles, which led to a better encapsulation particularly in the case of the utilized nucleic acids. This approach also creates the further potential for formulating a multifunctional delivery system. The novel approach of starch-chitosan-based complex-coacervation suggested in this study is a straightforward and promising technique to prepare versatile carrier systems with potential in nanomedicine applications. Therefore, we undertook preliminary studies of design, synthesis, and formulation of such carrier systems, and explored their flexibility and capacity for encapsulating selected model macromolecular drugs.

## 2. Experimental Section

### 2.1. Materials

As raw material, partially hydrolyzed potato starch (*M*_w_ of 1300 kDa), which was a kind gift from AVEBE (Veendam, The Netherlands), was used. Selective oxidation of the primary alcohol on starch was performed to increase water solubility and obtain an anionic charge. The oxidation procedure and molecular weight fractionation of three *M*_w_ samples (5, 30–100, and >100 kDa) was conducted in accordance with the protocol of Yamada et al. [12]. The obtained starch derivatives had an oxidation degree of 45%. The *M*_w_ fraction >100 kDa is used unless stated otherwise and is termed “anionic starch” in all further descriptions.

Chitosan oligosaccharide lactate (oligochitosan; *M*_w_ 5 kDa), polyvinyl alcohol (PVA; Mowiol^®^ 4-88), sodium hydroxide, trifluoroacetic acid (TFA), acetonitrile and acetic acid were purchased from Sigma-Aldrich (Darmstadt, Germany). Tobramycin sulfate salt and colistin sulfate salt were used as received also from Sigma-Aldrich. Ultrapure chitosan chloride salt (Protasan UP CL113; *M*_w_ ~90 kDa, deacetylation degree 75–90%) was obtained from FMC Biopolymer AS NovaMatrix (Sandvika, Norway). Purified water was produced by a Milli-Q water purification system from Merck Millipore (Darmstadt, Germany). O-Phthalaldehyde (OPA), 2-mercaptoethanol, phosphotungstic acid (PTA) and boric acid were used as purchased from Sigma-Aldrich.

Agarose SERVA for DNA Electrophoresis of research grade was bought from Serva (Heidelberg, Germany). Ethidium bromide solution (10 mg/mL), heparin sodium salt from porcine intestinal mucosa, 3-(4,5-dimethylthiazol-2-yl)-2,5-diphenyltetrazolium bromide) (MTT reagent), Triton™ X-100, dimethyl sulfoxide (DMSO) and Dulbecco’s phosphate buffered saline solution (PBS) were obtained from Sigma-Aldrich. Gibco Hanks’ balanced salt solution (HBSS) buffer was purchased from Thermo Fisher Scientific (Darmstadt, Germany). A549 cells (human lung carcinoma cell line, No. ACC 107) were obtained from DSMZ GmbH (Braunschweig, Germany). Cell culture medium (RPMI 1640) was purchased from PAA laboratories GmbH (Pasching, Austria) and supplemented with 10% fetal calf serum (FCS, Sigma-Aldrich). Plasmid DNA (pDNA) encoding for the fluorescent protein AmCyan was bought from Clontech Laboratories, Inc. (pAmCyan 1-N1, Mountain View, CA, USA). The plasmid was propagated in *Escherichia coli* DH5α and isolated with Qiagen EndoFree Plasmid Mega Kit (Qiagen, Hilden, Germany) to obtain pDNA of cell culture quality. jetPRIME^®^ transfection reagent was purchased from Polyplus-transfection (Illkirch, France). Rhodamine *Ricinus communis* agglutinin I (RGA I) was obtained from Vector Laboratories. 4′,6-diamidino-2-phenylindole (DAPI) was purchased from Life Technologies (Darmstadt, Germany).

### 2.2. Preparation, Optimization and Characterization of Starch–Chitosan Core Polyplexes

#### 2.2.1. Preparation and Optimization of Starch-Chitosan Core Polyplexes (CP)

Starch-chitosan core polyplexes (CP) were prepared by self-assembly of anionic starch derivatives and chitosan derivatives in aqueous medium. CP characteristics, including their: (i) surface properties; (ii) size; and (iii) physicochemical stability were varied by: (i) the molecular weight of utilized anionic starch and chitosan derivatives; (ii) polymer concentration; and (iii) molar ratio of carboxylate (COONa) to amine (NH_2_) groups (C/N ratio) in oxidized starch and chitosan, respectively. The polyplex formulation procedure is described in Scheme 1A. Briefly, a solution of anionic starch was prepared in Milli-Q water at a defined concentration, while the utilized chitosan derivative was solubilized in 0.02 M acetic acid, followed by pH adjustment to 5.5. The assembly into CP of oxidized starch and its counter excipient occurred by the addition of an appropriate amount of starch polymer solution into the pre-warmed solution of chitosan derivative, followed by 2 min of vortexing and 1 h incubation at room temperature. To prepare anionic core polyplexes (anCP), anionic starch (*M*_w_ of >100 kDa) and oligochitosan (*M*_w_ of 5 kDa) were employed at various C/N ratios, ranging from 50:1 to 10:1 and further to 1:1, designed to optimize the formulation and stability of the polyplexes. Cationic core polyplexes (cationic CP) were prepared by co-assembly of negative starch (*M*_w_ of 30–100 kDa) and Protasan (*M*_w_ of 90 kDa) having a higher amount of positively charged amine groups. The optimal C/N ratio was identified by investigating the ratios of 1:30, 1:10 and 1:1. All samples with a solvent pH-value of 5.5 were characterized by dynamic light scattering (DLS), using a Zetasizer Nano from Malvern Instruments (UK) to obtain hydrodynamic size, polydispersity index (PDI), and using laser Doppler velocimetry to obtain ζ-potential. All samples were prepared at least in three different batches.

#### 2.2.2. Preparation and Optimization of Protasan Coated CP (cCP)

Another approach taken to further improve the loading capacity of starch-chitosan carriers was to prepare coated polyplexes with a further layer of Protasan on anCP. The optimized coating method is described briefly as following: anCP were prepared as described and then coated with an additional layer of positively charged Protasan, by an association of amine functional groups of the chitosan and the anionic surface of the anCP (Scheme 1B). The coating solution was prepared by dissolving 3 mg of Protasan in 1 mL PVA 2% (*w/v*) solution, which was then diluted with Milli-Q water to a 1.5 mg/5 mL concentration for coating. A 500 µL volume (6.6 mg/mL) of anCP was added dropwise to the prepared Protasan solution, which was continuously stirred for 30 min at 150 rpm. This was followed by incubation at room temperature for 3 h prior to characterization. The resulting Protasan-coated anCP (cCP, *c* = 0.87 mg/mL) were kept for further studies. Samples were prepared in at least three different batches. All particle samples were characterized for their hydrodynamic size, PDI and ζ-potential. This method was also applied to investigate the physicochemical stability of anCP and cationic CP under storage conditions of 4 °C for 27 days.

#### 2.2.3. pH-Stability of Drug-Free CP and cCP

The colloidal stability of anCP and cCP at different pH values was investigated by incubating particle suspensions at pH values of 3.5, 4.0, 4.5, 5.5, 6.0, 7.5 and 8.0, all within the physiologically-relevant range. Samples were analyzed to obtain hydrodynamic size, PDI, and ζ-potential, after predetermined incubation times (30 min, 1 h, 3 h and 24 h). The pH-value was adjusted following polyplex preparation at pH 5.5 (as described above) by using either 0.02 M acetic acid solution or 1 M NaOH solution. All experiments were conducted in triplicates with *n* = 3, and results expressed as mean ± standard deviation (SD).

#### 2.2.4. Morphology

The morphology of all produced polyplexes was visualized by transmission electron microscopy (TEM, JEM 2011, JEOL, St Andrews, UK). Before the TEM visualization, 8.7 µg/10 µL of polyplexes were added on a copper grid (carbon films on 400 mesh copper grids, Plano GmbH, Wetzlar, Germany) and incubated for 10 min to allow an adhesion of polyplexes to the surface. The excess was removed, and polyplexes were further stained with 0.5% (*w/v*) PTA to improve the contrast of TEM images.

#### 2.2.5. Cytotoxicity Study: MTT Assay

A549 cells were seeded in a 96 well plate at a density of 1 × 10^5^ cells per well, in 200 μL of RPMI cell culture medium supplemented with 10% FCS. Cells were grown for 4 days prior to the conduction of the assay to allow for approximately 95% cell confluency. On Day 4, CP and cCP samples were diluted with a suitable amount of RPMI medium (without FCS) to achieve test concentrations of 5, 10, 40, 70, 100, 200 and 500 µg/mL. Cells were then washed twice with 200 µL HBSS buffer (pH 7.4), and polyplex samples were added to cells in triplicate. Cells incubated with only RPMI medium were used as a negative control (determined to result in 100% cell viability) and cells treated with 1% Triton™ X-100 in RPMI medium were used as positive control (designated as 0% cell viability). All samples were incubated with cells for 4 h, on a horizontal shaker with careful shaking at 150 rpm at 37 °C and 5% CO_2_. Subsequently, the supernatant was removed, and cells were washed once with HBSS. Then, 200 µL of the MTT-reagent (5 mg/mL) in HBSS was applied to each well and further incubated for 4 h with gentle shaking. The supernatant was then removed and DMSO was immediately added to achieve cell lysis. Cells were incubated in DMSO for 15 min under careful shaking and protected from light. The absorbance of each well at 550 nm was then measured with a plate reader (Infinite^®^ 200 Pro, TECAN, Männedorf, Switzerland). The percentage of viable cells was calculated in comparison to negative and positive controls as described by Nafee et al. [27]. 

### 2.3. Cationic Anti-Infective Loaded anCP

#### 2.3.1. Preparation and Optimization of Cationic Anti-Infective Loaded anCP

##### Isothermal Titration Calorimetry

**Scheme 2:**
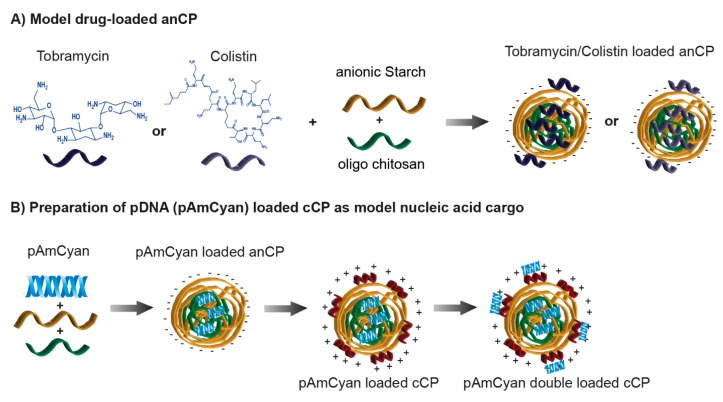
Illustration of starch-chitosan polyplex-preparation for drug-loaded polyplexes.

Two relevant anti-infectives were used to test the loading capacity of anCP. Tobramycin was used as an example of a cationic small molecule antibiotic having a molecular weight of 467.5 Da, and colistin (polymyxin E) was used as an example of a peptide antibiotic with a molecular weight of 1267.5 Da (Scheme 2A).

Interaction between anionic starch and the cationic anti-infectives tobramycin and colistin was investigated by isothermal titration calorimetry (ITC) using a NanoITC 2G (TA Instruments, New Castle, DE, USA). The purpose of such measurement was to optimize excipient to cargo ratio in drug loaded carrier production. Briefly, all drug and anionic starch solutions were prepared in milli-Q water. A 25 mM solution of tobramycin or colistin was prepared in a 250 µL syringe and used to saturate 1.5 mL of anionic starch at a concentration of 0.1 mM filled in the sample cell. Following an initial delay of 300 s, 250 µL of drug solution was repeatedly injected into the sample cell with a spacing of 500 s between injections, and at a reference power of 10 µCal/s. The final thermogram and thermodynamic parameters were produced by subtracting the heat of dilution of either tobramycin or colistin (25 mM in 1.5 mL milli-Q water), followed by fitting using the One Set of Sites model in the data analysis software NanoAnalyze. The free energy of binding (∆*G*) was calculated using the equation ∆*G* = ∆*H* − *T*∆*S*, where ∆*H* is the enthalpy change, *T* is temperature (Kelvin), and ∆*S* is the change in entropy. All measurements were performed at 25 °C.

##### Preparation and Optimization of Cationic Anti-Infective Loaded anCP

Both tobramycin and colistin were loaded using the same procedure, during formation of anCP, employing various C/N ratios, as follows: (i) 1 mg tobramycin or 3 mg colistin was incubated with an appropriate amount of anionic starch solution for 2 h; and (ii) pre-warmed chitosan solution at pH 5.5 was added, and coacervation was achieved by vortex mixing (2 min). 

The anti-infective loaded anCP suspension was then centrifuged at 13,000× *g* and 4 °C for 20 min at least twice and allowed to equilibrate at 4 °C overnight before conducting further experiments. In all experiments the supernatant produced by centrifugation was collected for drug loading quantification.

##### Loading Quantification

The degree of anti-infective loading in anCP was determined using an indirect quantification method (drug amount inside anCP = initial drug amount − drug amount in the supernatant). Colistin was quantified by high-performance liquid chromatography (HPLC), while tobramycin was quantified based on a protocol for detection of aminoglycosides [28], as detailed below.

##### HPLC Analysis

The HPLC analysis was performed on a Dionex UltiMate 3000 system (Thermo-Fischer Scientific, Dreieich, Germany) equipped with LPG-3400 SD pump, WPS-3000 autosampler, DAD3000 detector, and TCC-3000 column oven. Chromeleon software (Chromeleon 6.80 SP2 build 9.68, Thermo Scientific Dionex, Dreieich, Germany) was used for data analysis. A column set of LiChrospher^®^ 100 RP-18 (5 µm) LiChroCART^®^ 125-4, consisting of a 125 mm × 4 mm LiChrospher 100/RP-18 column (Merck-Hitachi, Darmstadt, Germany) with a LiChrospher 100/RP-18 guard column (5 μm) (Merck-Hitachi, Darmstadt, Germany) at 30 °C was used as stationary phase for all substances. A gradient method was used starting with 20% A, increasing to 50% A within 2 min, and holding for 1.5 min (A = acetonitrile, B = 0.1% TFA solution in water). Before injection, the samples were filtered through a cellulose acetate 0.2 μm membrane. The flow rate was 1.0 mL/min, and the injection volume was 50 μL. A calibration curve was constructed using eight different concentrations of colistin in water, ranging from 0.2 mg/mL to 0.005 mg/mL (*r*^2^ = 0.9955). All 8 standards were measured 5 times, and a percent relative standard deviation (% RSD) of less than 3.9% was calculated. The run time was 6 min, and a retention time of 3.6 min and 3.9 min was observed for colistin A and colistin B, respectively. As colistin is a mixture of two main fractions, colistin A and colistin B, both were quantified to determine colistin loading. The detection wavelength was 210 nm for colistin A and 214 nm for colistin B.

##### Aminoglycoside Detection Protocol

The product fluorescence of tobramycin reacted with a fluorescent reagent was measured at 344/450 nm (*E*x/*E*m) using a Tecan microplate reader following a published method [28]. To prepare the reagent solution, a 0.2 g amount of OPA reagent was dissolved in a mixture of 1 mL methanol, 19 mL boric acid 0.4 M at pH 10.4, and 0.4 mL of 14.3 M 2-mercaptoethanol. A 2 mL of the resulting mixture was then diluted with 16 mL methanol before use. A calibration curve was constructed using five different concentrations of tobramycin in water (0.04–0.005 mg/mL, *r*^2^ = 0.9976).

In both cases, the encapsulation efficiency (EE) and the drug loading rate (LR) were calculated according to the following equations:

(1) $$\mathrm{EE}=\;\frac{\mathrm{Weight}\;\mathrm{of}\;\mathrm{encapsulated}\;\mathrm{drug}\;\mathrm{in}\;\mathrm{nanoparticles}}{\mathrm{Initial}\;\mathrm{amount}\;\mathrm{of}\;\mathrm{drug}\;\mathrm{in}\;\mathrm{the}\;\mathrm{system}\;}\;\times \;100\;\mathrm{LR}=\;\frac{\mathrm{Weight}\;\mathrm{of}\;\mathrm{drug}\;\mathrm{in}\;\mathrm{nanoparticles}}{\mathrm{Weight}\;\mathrm{of}\;\mathrm{nanoparticles}\;}\;\times \;100$$

where “weight of nanoparticles” was calculated as weight of polymeric material + weight of encapsulated drug in nanoparticles.

Each sample was assayed at least in triplicate, and results are reported as the mean ± SD.

##### Drug Release Study

Tobramycin or colistin release profiles from tobramycin loaded anCP or colistin loaded anCP was performed in PBS (pH 7.4) at 37 °C. Briefly, either tobramycin loaded anCP or colistin loaded anCP was diluted in PBS to have final tobramycin or colistin concentration at 10% (*w/w*) and loaded into dialysis membrane (MWCO 1 kDa, Spectrum Labs, Rancho Domiguez, CA, USA) in the case of tobramycin, or dialysis membrane (MWCO 3.5–5 kDa, Spectrum Labs, USA) in the case of colistin. After that, the whole system was put into 20 mL PBS and placed on a shaker at 400 rpm at 37 °C. The concentration of released drug was analyzed by collecting samples from the supernatant during the period from 1 h to 24 h. The amount of colistin and tobramycin were determined by HPLC and aminoglycoside detection protocol, respectively. The volume was kept constant by refilling with an identical volume of PBS. The cumulative released drug (%) was calculated (mean ± SD of *n* = 3). Three independent experiments were conducted in triplicates, and results expressed as the mean ± standard deviation (SD).

#### 2.3.2. Minimum Inhibitory Concentration (MIC) Assay

The antimicrobial properties of anCP, anti-infective loaded anCP, and free drugs were performed by standard microbroth dilution assays with *Escherichia coli* (DH5α) and *Pseudomonas aeruginosa* (PA14) in 96 well plates. A suspension of *E. coli* or *P. aeruginosa* prepared from mid log cultures in Mueller-Hinton broth or Lysogeny Broth medium (at 25 °C) was first diluted to OD_600_ (absorption at 600 nm) 0.01, which corresponds to approximately 5 × 10^6^ CFU/mL (CFU, colony-forming units). Polyplex samples (anCP, drug-loaded anCP), free drug solution and PBS as control were then added to bacteria-containing wells by serial dilution over a range of 0.03–64 μg/mL. After incubation for 16 h at 37 °C, inhibitory concentration (IC) IC_90_ values were determined by sigmoidal curve fitting of absorption values (600 nm) that were measured on a Tecan microplate reader. The experiments were conducted in duplicate.

### 2.4. Preparation of pDNA Loaded cCP 

Plasmid DNA pAmCyan was incorporated into the polyplexes to evaluate the potential of the carrier system with respect to nucleic acid actives. A ratio of amine groups (chitosan) to phosphate groups (pDNA) of 20/1 was chosen and is referred to as N/P ratio. The preparation was performed in three steps: first, an appropriate amount of pAmCyan was added to a solution of anionic starch and mixed thoroughly. A 1 mL volume of this pAmCyan-starch solution was added to 1 mL of oligochitosan solution (650 μg/mL) and mixed immediately by vortex for 15 s. A further incubation for 1 h at room temperature was then carried out, leading to the formation of pAmCyan-loaded anCP. In the second step, the pAmCyan loaded anCP were coated by Protasan as described in Section 2.2, to form pAmCyan-loaded cCP. In the third step, a further layer of pAmCyan was applied to pAmCyan-loaded cCP (1:30 *w/w*) resulting in pAmCyan double loaded cCP (Scheme 2B). The pDNA encapsulation efficiency of each step was analyzed by pelleting the samples down and measuring the absorbance of unbound pDNA (at 260/280 nm with NanoDrop Spectrophotmeter) remaining in the supernatant after centrifugation for 30 min at 24,400× *g*. Thus, the amount of bound pDNA was examined indirectly. The products of each step were characterized to obtain hydrodynamic size, PDI, and ζ-potential, and their morphology was observed by TEM. 

#### 2.4.1. Determination the Complexation of pAmCyan in Starch-Chitosan Polyplexes 

Complexation and stability of pAmCyan in starch-chitosan polyplexes was evaluated by a gel retardation assay using agarose gel electrophoresis. Further, to facilitate DNA fragmentation, the endonuclease BamHI was used, which linearizes the plasmid, and heparin addition to cause the release of pDNA from the complex. Polyplexes containing 500 ng of pDNA per sample from each step of the formulation process were first digested with 0.5 µL BamHI for 2 h at 37 °C with shaking. Afterward, 3 µL (30 mg/mL) heparin was added to solutions of digested polyplexes, incubated for 15 min at room temperature and then mixed with 2 µL of orange DNA loading dye (6×; Thermo Fisher Scientific, Waltham, MA, USA). These mixtures were then loaded into 0.75% (*w/v*) agarose gel containing 5 µL of ethidium bromide and run for 60 min at 50 V in 0.5× TBE-buffer. The visualization of the bands was performed with a UV illuminator, Fusion FX7 imaging system from Peqlab (Erlangen, Germany). 

#### 2.4.2. In Vitro Transfection Studies in A549 Cells

To test the efficiency of the pAmCyan loaded polyplexes, in vitro transfection studies were performed in A549 cells. Briefly, A549 cells were seeded in 24-well plates, at a density of 25 × 10^4^ cells per well in 500 µL of RPMI cell culture medium with 10% FCS. Cells were grown for 2 days to reach a cell confluency of 60–70%. Polyplexes of the pAmCyan double loaded carrier system (see Section 2.4) containing 1 µg of pAmCyan (polyplex concentration ~60 μg/mL) were prepared with a ratio of 1:30, 1:50 and 1:100 between pDNA:polyplexes in 500 µL of HBSS buffer. Then, cells were washed twice with HBSS buffer and incubated with the polyplexes for 6 h. After 6 h of incubation, polyplexes were removed and replaced with RPMI containing 10% FCS. Cells were further grown for 2, 3 and 4 days to identify the time point of maximum reporter gene expression. For comparison, the commercially available transfection reagent jetPRIME^®^ was used as positive control. Cells treated with pAmCyan-free cCP and cell culture medium alone were used as negative controls. For confocal laser scanning microscope (CLSM; Leica TCS SP 8, Leica, Wetzlar, Germany) visualization, cell membranes were stained using RGA I (15 µg/mL), and cell nuclei were stained with DAPI (0.1 µg/mL). Samples were then fixed with 3% paraformaldehyde and stored at 4 °C until analysis. All images were acquired using a 25× water immersion objective at 1024 × 1024 resolution and further processed with LAS X software (LAS X 1.8.013370, Leica Microsystems, Leica, Germany). The percentage efficiency of transfected cells was quantified using flow cytometry (BD LSRFortessa™ Cell Analyzer, Biosciences, Heidelberg, Germany). Fifty thousand cells per sample were counted by the cytometer and data were analyzed using FlowJo software (FlowJo 7.6.5, FlowJo LLC, Ashland, OR, USA). Three independent experiments were performed in triplicates, and results expressed as the mean ± standard deviation (SD).

## 3. Results and Discussion

### 3.1. Preparation and Characterization of Drug-Free Starch-Chitosan Polyplexes

**Figure 1:**
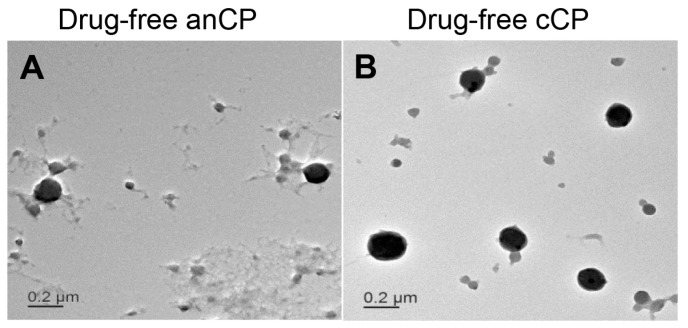
Transmission electron microscope (TEM) images of drug-free starch-chitosan polyplexes stained by 0.5% phosphotungstic acid solution: (**A**) drug-free anCP; and (**B**) drug-free cCP.

This study represents an extension in comparison to the particle preparation approach of Barthold et al. [29], in which the large poly dispersity index modified starch was employed for colloidal formation. Furthermore, the chemical modification in that reported study, which was used to produce cationic starch derivative, resulted in unfavorably additional synthesis step. Although the particle preparation was well established, the lack of cationic strength due to an obviously low converting yield of cationic starch synthesis limited the carrier capacity for anionic net charge actives of such system. Thus, we used fractionally modified starch derivatives to have better control of colloidal stability, and different molecular weight chitosan derivatives as strong counter excipient for the polyplexes produce. Both excipients are polysaccharides and therefore have favorable characteristics with respect to biological safety, biocompatibility and biodegradability. The simple production of polyplexes using these excipients has the perspective to be readily up-scaled. In the first series of preparations, we studied the plain polymeric complexes by combining both excipients in aqueous solution, with the electrostatic interaction between opposite charges of the individual polymers resulting in polyplex self-assembly. During the optimization of this process, various combinations of types of polymers, C/N ratio, and initial polymer solution concentration were investigated to find a stable and narrow size distribution of the produced colloidal structures (details of the optimization can be found in the Supplementary Materials, Tables S1 and S2). The best of several stable polyplex formulations was produced using a C/N ratio of 10:1, utilizing anionic starch and oligochitosan. Starch-chitosan polyplexes were obtained with an anionic surface charge evidenced by a ζ-potential of around −30 mV. The size of polyplexes could be varied from 150 nm to 350 nm by changing of polymer concentration, with a narrow PDI (<0.3) in all cases. The impact of polymer concentration on polyplex size was expected and already described for comparable systems [30,31]. Spherical polyplex morphology was visualized using TEM (Figure 1A). 

Reversing the C/N ratio to 1:10, and using starch (*M*_w_ 30–100 kDa) and Protasan (*M*_w_ ~90 kDa) resulted in a switch of the surface charge from anionic to cationic (further termed as cationic CP), with a ζ-potential of around +40 mV. The size of particles varied from 214.3 nm to approximately 400 nm depending on the polymer concentration and C/N ratio (Supplementary Materials, Tables S1 and S2). As both anCP and cationic CP systems formed as a result of attractive forces of polymer functional groups, further aggregation of systems over time may potentially occur; the physical stability of the polyplexes was therefore studied over a time course with storage at 4 °C. The colloidal characteristics of both, anCP and cationic CP, remained stable for 27 days with a PDI of ~0.18 and a ζ-potential of −30 mV and +35 mV for anCP and cationic CP, respectively (Supplementary Materials, Figure S1). Consequently, the utilized preparation process represents a straightforward approach for the formulation of versatile nanoparticles. 

The possibility to control the surface charge of a nanocarrier is advantageous for both improving the interaction with the drug to be encapsulated as well as in a later stage the interaction with the target cell [32]. Therefore, the ability to tune surface charge by changing the C/N ratio and molecular weight of starch and chitosan derivatives is a distinct advantage of this novel type of carrier. The ability to load drug molecules of differing structure size and charge, such as e.g., low-*M*_w_ anti-infectives as well as high-*M*_w_ plasmid DNA, into these carriers was then investigated. Furthermore, a simple coating process was employed to minimize the use of cationic polymer, while still allowing for positive surface charge tuning of particles. The anCP were coated with an additional layer of Protasan (*M*_w_ of 90 kDa) resulting in cationic coated polyplexes, cCP. The organic solvent-free procedure was performed in aqueous solution in the presence of PVA as a stabilizer, and led to stable cationic particles with a ζ-potential of +27.1 mV, and a spherical morphology (Figure 1B). The hydrodynamic size and PDI decreased in comparison to anCP (Table 1, Supplementary Materials Table S3) due to the improved electrostatic interaction between the excipients. Furthermore, the anionic, cationic and coated polyplexes overall indicated ζ-potential values of around ±30 mV at which the value ensures improved colloidal stability [33,34,35], giving the polyplexes the possibility to survive and overcome various biological barriers and reach a specific site of interest.

#### 3.1.1. Colloidal Stability of Drug-Free anCP and cCP

To explore the potential to administer anCP and cCP by various routes, the physicochemical stability of these systems was investigated at pH values ranging from 3.5 to 8.0, as relevant for various drug administration pathways. The stability of the polyplexes in different conditions of pH was investigated following 30 min, 1 h, 3 h and 24 h of incubation. As the assembly of the polysaccharide nanoparticulate systems was based on electrostatic interaction, stability of such systems mainly depends on its surface properties which are, in turn, influenced by surrounding environmental factors, e.g., ionic strength and pH values [35,36]. anCP showed stable characteristics regarding size, PDI and ζ-potential, even at the lowest investigated pH value of 3.5 after 3 h incubation (Supplementary Materials, Figure S2). In agreement with the results of Yamada et al. [12], the relatively high *M*_w_ (>100 kDa) of the anionic starch clearly aids in stabilization of the particles.

**Figure 2:**
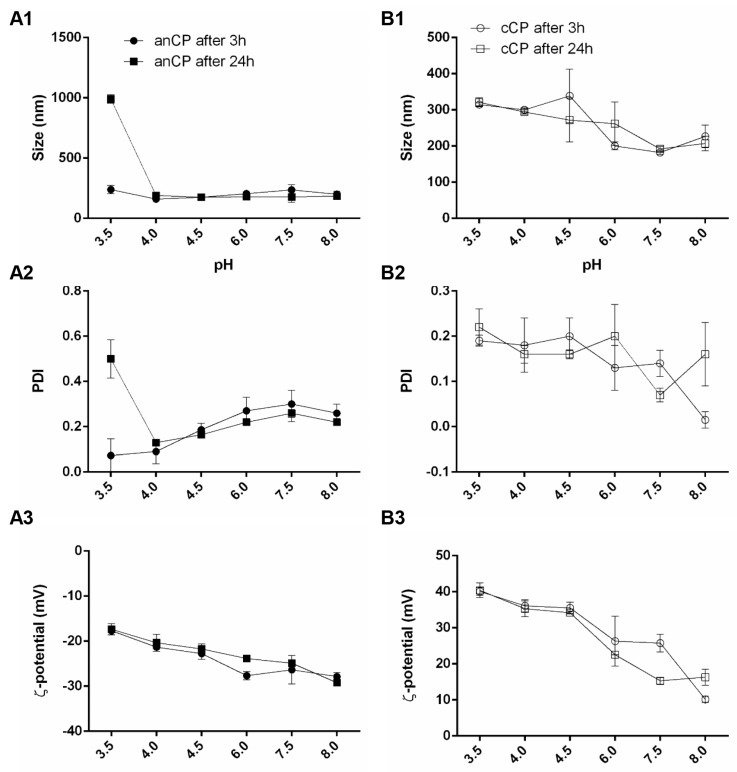
Characteristics of anionic core polyplexes (anCP, **A1**, **A2** and **A3**) and Protasan-coated core polyplexes (cCP, **B1**, **B2** and **B3**) after 3 h and 24 h incubation in pH conditions ranging from 3.5 to 8.0. The pH-values changed accordingly starting from an initial pH-value of the samples of 5.5. *n* = 3, mean ± SD.

However, the possible dissociation of carboxylate groups on particle surfaces may have eventually led to colloidal aggregation [36] at pH 3.5 and hence destabilized the polyplexes, as indicated by stability data after 24 h of incubation. By contrast the anCP remained stable at all other, higher, pH values after a 24 h incubation (Figure 2), which could be explained by an enhanced repulsive force among anionic particles due to increasing deprotonation of surface carboxylate groups at high pH values. The stability test performed on cCP revealed a stable particle size and PDI at all pH values after 24 h, however a reduction in cCP ζ-potential was seen from pH 3.5 to 8.0 (Figure 2). This behavior is explained by protonation of chitosan molecules, which, being a weak polyelectrolyte with a p*K*_a_ of approximately 6.5, has a changing protonation degree depending on the pH of the surrounding solution [37]. An increase in pH value up to 8.0 resulted in a diminishing protonation degree of the chitosan polymer [37,38], thereby resulting in a decrease ζ-potential. Nevertheless, a continued stability of cCP at all tested pH values, especially at pH 7.5 and 8, which are higher than the chitosan p*K*_a_ value, could be conferred by the presence of PVA, as a stabilizer that interrupts colloid interaction and aggregation. The stability of both anCP and cCP over a broad range of pH values clearly indicated flexibility in the potential application of such a tunable carrier system, for drug delivery via various routes of administration. The system could be considered for use in pulmonary delivery, where the local pH is nearly neutral; for gastrointestinal and vaginal delivery, where a low pH environment is encountered [39,40,41]; and in other applications.

#### 3.1.2. Cytotoxicity Assessment

**Figure 3:**
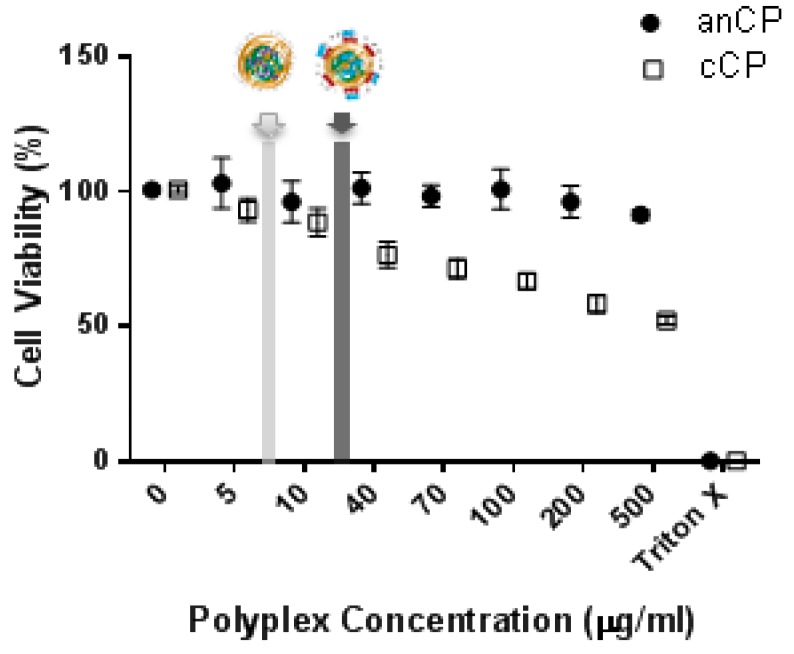
Cell viability assayed by MTT after 4 h incubation (mean ± SD, *n* = 9 from three independent experiments).

A549 cells were used in our study as a model cell line to test the potential of our carrier system. Figure 3 shows the viability of A549 cells exposed to anCP and cCP with concentrations up to 500 µg/mL, with the light grey area marking the concentration used for later MIC assays and the dark grey showing the concentration employed in subsequent transfection studies. The anCP demonstrated almost no cytotoxicity over the tested concentration range, with an observed cell viability of nearly 100% at all concentrations. However, in contrast, cell viability decreased markedly following treatment with increasing concentrations of cCP. This may be due to their cationic surface charge [42], which, on the other hand, could potentially facilitate a higher cellular uptake of cCP [43], as is particularly relevant for pDNA delivery applications. Ultimately, the cationic surface charge of such pDNA polyplex must be carefully tuned towards an acceptable compromise between transfection efficacy and biocompatibility.

### 3.2. Loading of anCP with Low-Mw Anti-Infectives

#### 3.2.1. Optimization of the Preparation Process and Drug-Loading

**Figure 4:**
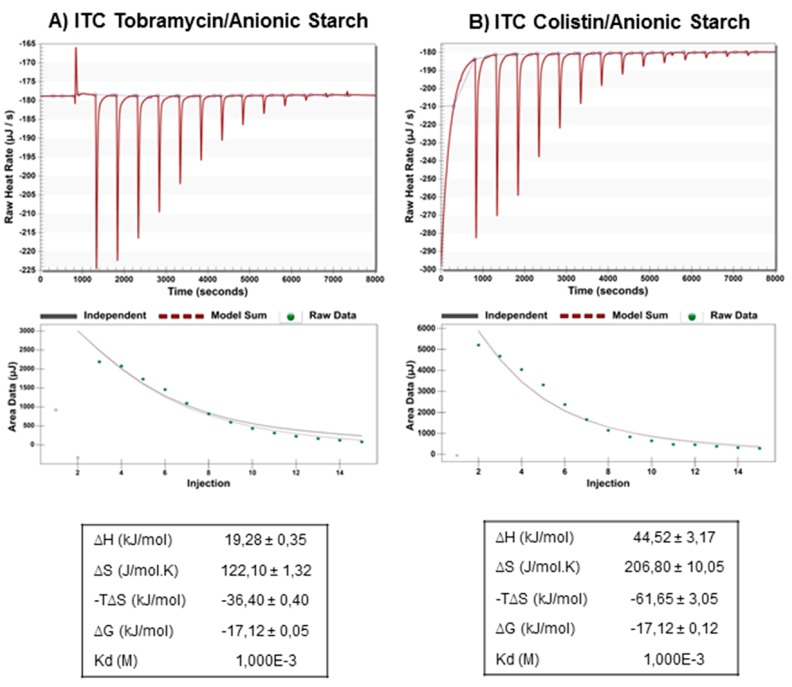
Isothermal titration calorimetry (ITC): (**A**) titration of tobramycin (25 mM) into anionic starch derivative (*M*_w_ of >100 kDa) (0.1 mM); and (**B**) titration of colistin (25 mM) into anionic starch derivative (*M*_w_ of >100 kDa) (0.1 mM).

To explore the potential of anCP as a carrier for diverse drug cargos, the aminoglycoside tobramycin (*M*_w_ = 467.5 Da), and the oligopeptide colistin (*M*_w_ = 1267.5 Da) were chosen as low molecular weight cargos. Tobramycin and colistin are active against Gram negative bacteria, and are two of the four drugs specifically approved in Europe for application as inhaled therapies for chronic bronchopulmonary *P. aeruginosa* infection in cystic fibrosis patients [25,26,44]. Fast elimination and poor permeability however often limit the delivery of hydrophilic anti-infectives, such as tobramycin and colistin, requiring frequent and high dosing with the risk of adverse drug effects and the development of bacterial resistance. Approaches to encapsulate these essential anti-infectives within drug carrier systems to avoid such delivery problems and preserve their activity have therefore been described [30,45]. Tobramycin and colistin both have net positive charges at a neutral pH value due to the presence of amine functional groups in their structures. Thus, it was hypothesized that their properties would be conducive to incorporation into anionic starch-based particles. Consequently, the interaction of further applied chitosan molecules and anionic starch would be affected, which would eventually lead to unstable colloids and aggregation of the resulting system. Therefore, before coacervation, the potential binding of anti-infective molecules to oxidized starch polymer (*M*_w_ of >100 kDa), was investigated by isothermal titration calorimetry (ITC), which revealed the thermodynamics of the binding and helped to estimate the optimal drug amount for loading in anCP. Tobramycin and colistin respectively were injected as aqueous solutions to saturate an anionic starch solution, as shown in Figure 4. Values in the inset tables were calculated by the software NanoAnalyze, yielding the same Gibbs free energy (∆*G*) for the interaction of around −17.12 kJ/mol for both tobramycin and colistin respectively with the anionic starch polymer. Moreover, based on the thermograms from the ITC analysis, the amount of tobramycin or colistin needed to completely saturate the anionic starch polymer is known. To completely saturate the fixed amount of anionic starch (e.g., 5 mg), there is a need of 1 mg tobramycin, while the needed amount of colistin is 3 mg. The interaction between drug molecule-anionic starch, as well as the number of amine groups on each drug molecule are similar; their molecular weight, however, are nearly three times different. Thus, the amount of the used colislin was three times higher than that of tobramycin. With the aforementioned optimization, the amounts of drugs were selected and for further investigation of drug-loaded anCP.

Having illustrated a clear interaction of tobramycin and colistin with anionic starch, preparation of drug-loaded anCP using chitosan as a counter polymer was investigated. Anionic starch and the selected anti-infective were first incubated, followed by the addition of an appropriate amount of pre-warmed chitosan solution, leading to the formation of polyplexes by self-assembly of these polyelectrolytes. A comparable particle preparing procedure was described by Deacon et al. for tobramycin and alginate [30]. The C/N ratio and the initial concentrations of the three components (anionic starch, chitosan, and tobramycin or colistin) were varied, and the characteristics of the resulting polyplexes were investigated in order to achieve an optimal formulation. The results of this optimization work are highlighted in Table 2, with additional data shown in the Supplementary Materials (Tables S4 and S5).

**Figure 5:**
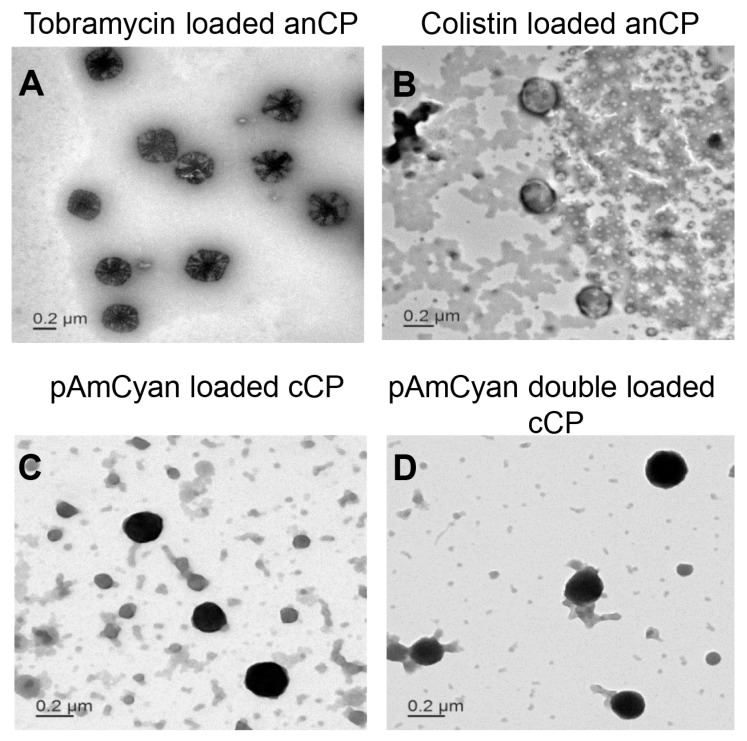
Transmission electron microscope (TEM) images of drug-loaded starch-chitosan polyplexes stained by 0.5% phosphotungstic acid solution: (**A**) tobramycin loaded anCP; (**B**) colistin loaded anCP; (**C**) pAmCyan loaded cCP; and (**D**) pAmCyan double loaded cCP.

The loading capacity of tobramycin and colistin in anCP was then evaluated. Encapsulation of these molecules into anCP was based on the association of anionic ions of oxidized starch and cationic ions of drug molecules. Hence, by using a fixed amount of drug molecules, and varying the C/N ratio (by varying the amount of added chitosan derivative) as well as the initial concentration of polymer solution, stable drug-loaded colloids could be produced. As shown in Table S4, tobramycin-loaded anCP ranging in size from 165.8 ± 0.8 nm to 375.9 ± 1.8 nm with a homogenous distribution (PDI < 0.3) were formed. The ζ-potential of tobramycin-loaded anCP generally increased from nearly −30 mV to average −17 mV, which suggested the presence of cationic drug molecules not only within the polyplex matrix, but also on the surface of polyplexes. An increasing C/N ratio also resulted in a tendency for decreasing particle size from 375.9 ± 1.8 nm to 175.2 ± 2.8 nm. This decrease in size could be due to a condensing effect when using a higher amount of starch, which introduced an excess of available anionic ions for interaction with chitosan, even after incubation with tobramycin. As a result, the colloidal characteristics of tobramycin-loaded nanoparticles were not significantly different to those of unloaded systems. To obtain colistin-loaded anCP, an amount of colistin three times higher in comparison to tobramycin was employed for pre-incubation with anionic starch, due to the molecular weight difference between the two drugs. Colistin-loaded anCP were prepared again using varying polymer concentrations. As the final concentration decreased, while C/N ratio was maintained at 40/1, the particle size decreased from 324.4 ± 3.6 nm to 266.3 ± 6.5 nm (Table S5). The colistin loaded systems were also stable and homogenous with PDI values lower than 0.3. The results, therefore, clearly show that a reduction in polyplex size resulted from a decrease in employed polymer concentration; this is also in accordance with observations in previous studies [31]. Overall, there was an increase in the ζ-potential of drug loaded carriers as compared to unloaded, which is evidence for the presence of positive net charge anti-infective molecules on carrier surfaces. The size of colistin-loaded anCP was generally larger than the corresponding tobramycin-loaded anCP, which could be explained by the possible formation of colistin micelles during incubation with starch solution. This is made possible by the amphiphilic molecular structure of colistin, which possesses a lipophilic fatty acyl tail and a hydrophilic head group [46]. Consequently, the addition of chitosan supported the colloidal stability of the polyplex system. The morphology of anti-infective loaded anCP was spherical as investigated by TEM (Figure 5A,B). The encapsulation efficiency (EE) and loading rate (LR) of colistin- and tobramycin-loaded anCP are highlighted in Table 2 and Tables S6 and S7. The EE and LR values were indirectly calculated by collecting supernatants after two washing steps. As determined using HPLC, colistin encapsulated within anCP showed maximum values of 96.57 ± 0.19% and 22.70 ± 0.33% for EE and LR, respectively. Incorporation of tobramycin, determined by product fluorescence at 344/450 nm, also showed an EE higher than 98% in all cases, but comparatively lower LR values (2.9 ± 0.0% maximum). The high EE of both model drugs (>90% in all cases) was a result of pre-determination of the interaction between drug molecules and anionic starch, which allowed estimating the amount of used drug in encapsulation and thereby maximization of the encapsulation efficiency. The LR of tobramycin-loaded anCP showed a rational loading capacity for polymeric nanoparticles with a size of approximately 200 nm, while the LR of colistin-loaded anCP was surprisingly high. This might be due to the aforementioned micelle formation of colistin molecules, stabilized by the starch polymer solution. Hence, colistin could be localized in the core of nanoparticles, covered by starch polymer molecules, and could also be loaded on the surface of the system due to charge interaction. The results clearly demonstrate the capacity of the anCP carrier system to be loaded with either type of low-*M*_w_ anti-infectives.

Furthermore, the cumulative release profile of both tobramycin and colistin from drug-loaded anCP were studied in PBS at 37 °C, the results are shown in Figure S3. Clearly, the controlled release of anti-infective in PBS could be observed in both cases, with over 40% and 20% of drug released over the period 16–24 h for tobramycin and colistin, respectively. The initial burst after 4–6 h incubation was recorded as on average nearly 30% for tobramycin and 20% for colistin. The percentage of initial anti-infective released from the anCP would represent the amount of drug molecule loaded on the particles surface. Interestingly, the release of colistin at all time points are relatively lower than that of tobramycin, which would again be explained by the aforementioned micelle formation of colistin molecules that are stabilized and maybe then embedded inside the polymeric polyplex. The release results would help predict the drug carriers behavior in further in vitro experiments. To evaluate the release of the anti-infectives from drug-loaded anCP, and have better insight into the controlled release in vitro or in vivo, in which other components exist, e.g., bacteria, would require more complex biologically simulated tests that were beyond the scope of the present study.

#### 3.2.2. Efficacy of Anti-Infective Loaded anCP

While anCP have the capacity to load different types of anti-infectives including small molecule and peptide drugs, it is important that the particle excipients do not interfere with action of active agents which might confound the further evaluation of drug delivery systems. Hence, the anti-microbial activity of blank anCP and anti-infective loaded anCP were studied against *E. coli* and *P. aeruginosa* in comparison to the use of free drugs. As shown in Table 3, the antibacterial activity of drug-loaded anCP was relatively similar to that of the corresponding free drug. MIC values obtained show that blank anCP were not active against *E. coli* and *P. aeruginosa* at the highest tested concentrations, which means the formation of polyplexes with anCP did not compromise the intrinsic anti-microbial efficiency of either antibiotic. To demonstrate a superior safety and efficiency profile of such nanocarriers in comparison to the free drug would require some more complex biological test systems that were beyond the scope of the present study. 

### 3.3. Loading of anCP with High-Mw pDNA

**Figure 6:**
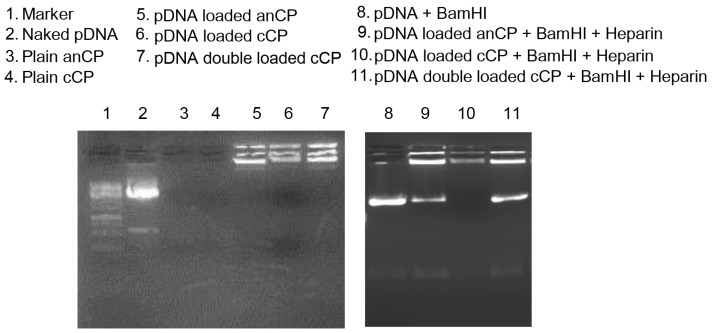
Gel retardation assay using agarose gel electrophoresis of plain and pDNA (pAmCyan) incorporated polyplexes for all three preparation steps in comparison with naked pDNA (undigested pDNA) and digested pDNA (pDNA + BamHI).

A model plasmid DNA encoding a fluorescent dye (pAmCyan) was further incorporated into the carrier system in a three-step procedure (core formation, Protasan coating and pDNA complexation), to demonstrate the ability of the polyplexes to deliver a broad spectrum of cargos. The three-step procedure also lead to an increase of nucleic acid encapsulation within the polyplexes, protecting nucleic acids from enzymatic degradation. Produced pAmCyan loaded polyplexes were again found to have a spherical structure (Figure 5C,D). The physiochemical characteristics of all intermediate and final polyplexes in this stepwise production can be found in Table 2. Each subsequent step in the preparation procedure results in a denser complexation, with the pAmCyan double loaded cCP showing the smallest size and most narrow size distribution (lowest PDI value). Furthermore, the ζ-potential was observed to switch from negative to positive after coating with Protasan, with a further slight decrease after complexation with negatively charged pAmCyan. The additional complexation with pAmCyan resulted in a 15% higher encapsulation efficiency in comparison to the intermediate step 2 (Table 2). Additionally, agarose gel electrophoresis (Figure 6, left) elucidates that no pDNA could run through the gel, which indicates that pDNA is strongly complexed within the polyplexes. Only further treatment with BamHI and heparin causes pDNA release as seen through the bands (Figure 6, right). Furthermore, pDNA loaded anCP and pDNA associated on the surface of polyplexes (pDNA double loaded cCP) allow an easier intercalation of EtBr and faster release with heparin, whereas pDNA loaded cCP is densely packed impeding pDNA release as no free pDNA bands can be observed in the gel.

#### Potential of Polyplexes for pDNA Delivery

**Figure 7:**
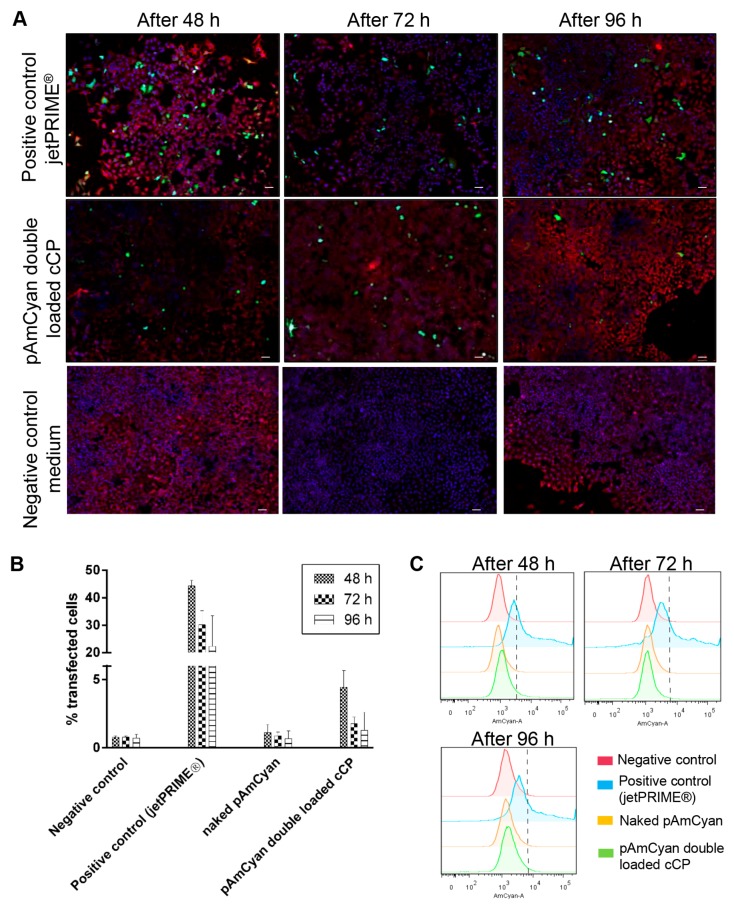
(**A**) Representative confocal images of A549 cells transfected with pAmCyan double loaded pAmCyan by using jetPRIME^®^ as positive control and only cell culture medium as negative control. Transfection was analyzed with CLSM after 48 h, 72 h, and 96 h. Green fluorescence reveals cells successfully transfected with the polyplexes while their morphology remains consistent with non-transfected cells (red: cell membrane; blue: cell nucleus; scale bar 50 μm). (**B**) The transfection efficiency was further quantified using flow cytometry, which indicated the highest amount of transfection after 48 h for pAmCyan double loaded cCP. (**C**) Representative graphs obtained with flow cytometer.

Using nanoparticles as a non-viral delivery system for gene therapy represents a significant challenge, as nanocarriers need to cross several biological barriers while preserving the functionality of carried pDNA. pDNA condensed inside the nanocarriers must survive the acidic conditions inside the lysosomes and escape the lysosomal compartment in order to cross the nuclear membrane [43]. Current knowledge of polymeric transfection systems suggests that a good pH-buffering capacity (a process known as the “proton sponge effect”) [47] is an important factor in the achievement of endosomal escape. Here, the potential of starch–chitosan polyplexes for nucleic acid delivery was explored by in vitro transfection studies using A549 cells. Three different ratios between pDNA:polyplexes have been studied to investigate the best transfection rate. While 1:50 and 1:100 show no significant transfection (data not shown), 1:30 mediated successful transfection, with the highest reporter gene expression observed after 48 h with 5% of transfected cells. In comparison, jetPRIME^®^ as positive control had a higher transfection efficiency (45%) after 48 h, which rapidly decreased to 30% after 72 h and to 25% after 96 h (Figure 7). The comparatively lower transfection efficiency of the polyplexes may be due to a high stability of condensed pDNA, leading to an incomplete release of pDNA inside the cytoplasmic compartment [48,49]. Further improvement of the transfection efficiency would presumably be achievable by addition of endosomal escape moieties [50,51], or with chitosan derivatives (e.g., trimethylation or amino acid conjugation) [52,53]. However, such efficacy improvements often impact the biocompatibility. Thus, optimization between safety and efficacy should be performed for a selected nucleotide type, target application, and delivery route, since carrier stability, cellular uptake, and functional efficacy are highly dependent on all these factors. 

## 4. Conclusions

In this work, we produced a flexible, straightforward and organic solvent-free procedure for the manufacture of nanocarrier systems based on the natural, biodegradable and biocompatible polysaccharides starch and chitosan. Starch and chitosan derivatives of different *M*_w_ ranges were combined by adjusting the molar ratio of carboxyl and amine functional groups, polymer concentration and counter polymer type to obtain a delivery system with tunable properties including surface charge and size. Core polyplexes (CP) were built by complex coacervation of anionic starch (*M*_w_ ~100 kDa) with positively charged chitosan derivatives (*M*_w_ ~5 kDa) in aqueous solution. The polyplexes with the best colloidal properties were obtained at a molar ratio of carboxyl and amine groups of 10:1. The negatively charged core polyplexes remained stable on storage for over 27 days. We further focused on optimizing anionic CPs by coating them with an additional layer of chitosan (Protasan, *M*_w_ ~90 kDa). Cell viability testing of anCPs and cCPs indicated a low level of cytotoxicity acceptable for use in biological systems, and colloidal stability at different tested pH values. The developed anCP system further showed good carrier properties, allowing for high encapsulation efficiency (>90%) of cationic peptide (colistin) and small molecule (tobramycin) anti-infectives without compromising antimicrobial activity. Moreover, the cationic polyplexes, cCP, allowed for double encapsulation of plasmid DNA (pAmCyan) for intracellular delivery as confirmed by gel retardation assay, and facilitating in-vitro transfection in A549 cells.

Starch-chitosan polyplexes show high flexibility for designing multifunctional carriers, in which for example the core polyplexes can encapsulate anti-infectives, while the outer coating layer could be used to incorporate other components like enzymes or nucleases (e.g., deoxyribonuclease I) to enhance drug penetration through biofilms or mucus [30]. For gene therapy purposes the inner polyplex can be used to carry and protect plasmid DNA, while the surface could be decorated with a second polynucleotide.

## Figures and Tables

**Table 1:** Summary of characteristics of representative drug-free (plain) polyplexes. All measurements were conducted in triplicates. *n* = 3, mean ± SD.

Polyplexes	Size (nm)	PDI	ζ-potential (mV)
Drug-free anCP	287.9 ± 5.0	0.22 ± 0.01	−29.7 ± 0.4
Drug-free cCP	205.4 ± 3.9	0.14 ± 0.02	27.1 ± 1.0

**Table 2:** Summary of characteristics of drug-loaded anCP, %EE = encapsulation efficiency and %LR = loading rate. All measurements were conducted in triplicates. *n* = 3, mean ± SD.

Polyplexes	Size (nm)	PDI	ζ-potential (mV)	%EE	%LR
Tobramycin loaded anCP	175.2 ± 2.8	0.18 ± 0.00	−16.8 ± 1.0	98.7 ± 0.1	2.9 ± 0.0
Colistin loaded anCP	266.3 ± 6.5	0.27 ± 0.01	−14.6 ± 0.5	96.6 ± 0.2	17.2 ± 0.1
pAmCyan loaded anCP	271.8 ± 2.4	0.25 ± 0.01	−29.8 ± 0.6	76.6 ± 0.6	0.3 ± 0.002
pAmCyan loaded cCP	214.0 ± 3.5	0.17 ± 0.01	28.0 ± 0.6	67.7 ± 14.1	0.2 ± 0.036
pAmCyan double loaded cCP	204.6 ± 3.5	0.16 ± 0.02	25.5 ± 0.6	93.9 ± 4.5	3.3 ± 0.150

**Table 3:** MIC assay results against *E. coli* and *P. aeruginosa.*

Samples	IC90 against *E. coli* (μg/mL)	IC90 against *P. aeruginosa* (μg/mL)
Tobramycin	0.2–0.3	1.56
Tobramycin loaded anCP	0.2–0.3 *	1.56 *
Colistin	0.4–0.5	3.125
Colistin loaded anCP	0.5 *	3.125–6.25 *
anCP	>64	>64
PBS buffer	no inhibition	no inhibition

* Drug content in anCP.

## Supplementary Materials

The following are available online at http://www.mdpi.com/2073-4360/10/3/252/s1, Figure S1: Physicochemical stability of starch-chitosan CP, in which anCP was produced with C/N ratio 10/1, and catCP was produced with C/N ratio 1/10, upon storage (4 °C). The particles were diluted into Milli-Q water at each time point for the measurement of size, PDI and ζ-potential. *N* = 3, *n* = 3, mean ± SD; Figure S2: Physicochemical stability of starch-chitosan anCP and cCP at different pH values ranging from 3.5 to 8.0, after 30 min and 1 h incubation. The initial pH-value of the samples was 5.5. *N* = 3, *n* = 3, mean ± SD; Figure S3: Cumulative release of tobramycin from tobramycin loaded anCP, and colistin from colistin loaded anCP performed in PBS at 37 °C. *N* = 3, *n* = 3, mean ± SD; Table S1: Summary of starch-chitosan CP characteristics obtained by varying polymer types, polymer concentration, and C/N molar ratio. *N* > 3, *n* = 3, mean ± SD; Table S2: Summary of starch-chitosan CP characterization with optimal C/N ratio varied by change of polymer concentration. *N* > 3, *n* = 3, mean ± SD; Table S3: Summary of anionic CP (anCP) and Protasan coated anCP (cCP) characteristics, in which anCP was produced with parameters, namely C/N ratio 10/1, and polymer concentration at 6.5 mg/mL. *N* > 3, *n* = 3, mean ± SD; Table S4: Summary of tobramycin-loaded anCP characteristics achieved by variation of C/N ratio and polymer concentration. *N* > 3, *n* = 3, mean ± SD; Table S5: Summary of colistin-loaded anCP characteristics resulting from variation of polymer concentration. *N* > 3, *n* = 3, mean ± SD; Table S6: Summary of drug loading quantification of tobramycin-loaded anCP. *N* > 3, *n* = 3, mean ± SD; Table S7: Summary of drug loading quantification of colistin-loaded anCP. *N* > 3, *n* = 3, mean ± SD.

## Abbreviations

CP	core polyplexes
anCP	anionic core polyplexes
cationic CP (or catCP)	cationic core polyplexes
cCP	coated polyplexes
