# Supplementary material for: Starch-Chitosan Polyplexes: A Versatile Carrier System for Anti-Infectives and Gene Delivery
Source: Polymers (Basel). 2018 Mar 1;10(3):252. doi: 10.3390/polym10030252 (PMC6415184; doi:10.3390/polym10030252)
Supplement: Supplementary file 1 [file polymers-10-00252-s001.docx]

Supporting information:

Starch-Chitosan Polyplexes: A Versatile Carrier System for Anti-Infectives and Gene Delivery

Hanzey Yasar ^1,2,†^, Duy-Khiet Ho ^1,2,†^, Chiara De Rossi ^1^, Jennifer Herrmann ^1^, Sarah Gordon ^1^, Brigitta Loretz ^1,^* and Claus-Michael Lehr ^1,2^

^1^ Helmholtz Institute for Pharmaceutical Research Saarland (HIPS), Helmholtz Center for Infection Research (HZI), Saarland University, D-66123 Saarbrücken, Germany

^2^ Department of Pharmacy, Saarland University, D-66123 Saarbrücken, Germany

† These authors contributed equally to this work.

**Abbreviations**

CP – core polyplexes, anCP – anionic core polyplexes, cationic CP (or catCP) – cationic core polyplexes, cCP – coated polyplexes

Table S1. Summary of starch-chitosan CP characteristics obtained by varying polymer types, polymer concentration, and C/N molar ratio. *N* > 3, n = 3, mean ± SD.


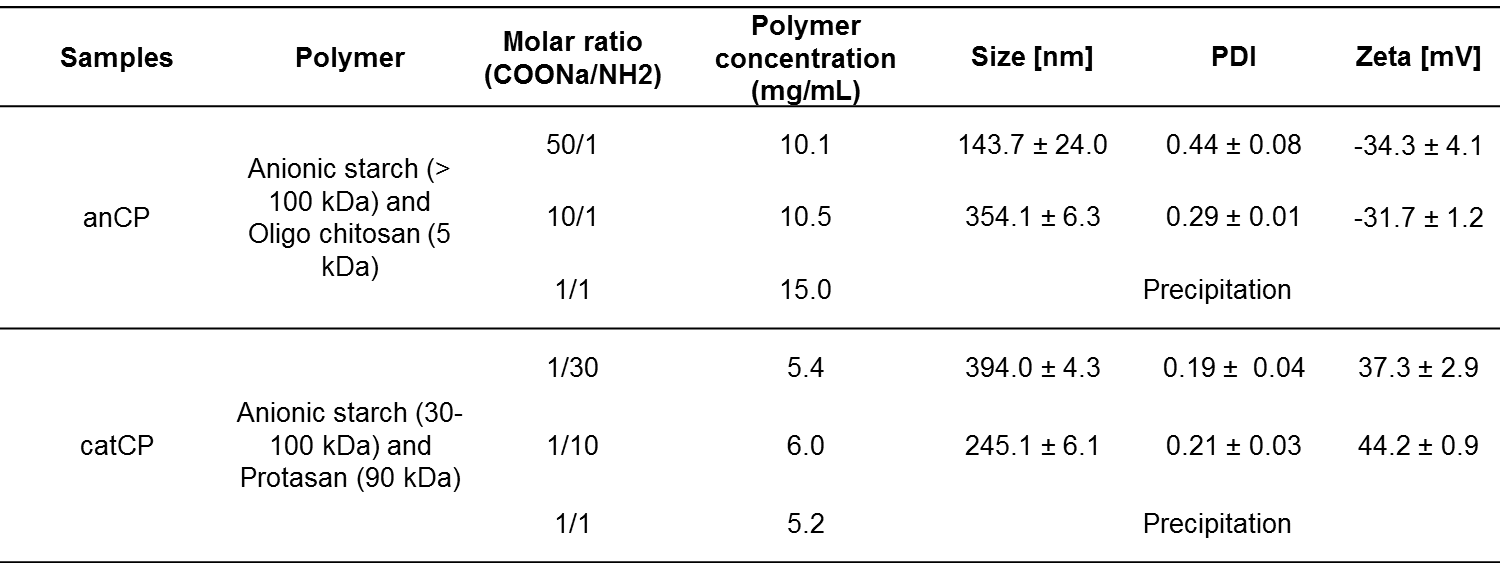


The anCP with C/N ratio 50/1 became precipitated after 3 days (data not shown) due to the low concentration of chitosan which might not be enough for the complexation and the resulted high polydispersity index under storage conditions (4 ^o^C). The catCP, in turn, could be formed with C/N ratio 1/30. However, the particles size had increasing tendency which is similar with that of catCP formed by C/N ratio 1/10 (data shown in Figure S1) and became precipitated after 5 days (data not shown). The observation would be explained by the adhesive nature of chitosan, and typical property of non-crosslinked polymeric polyplex system. Hence, the C/N ratio 10/1 and 1/10 were used to further experiment to produce anCP and catCP respectively.

Table S2. Summary of starch-chitosan CP characterization with optimal C/N ratio varied by change of polymer concentration. *N* > 3, n = 3, mean ± SD


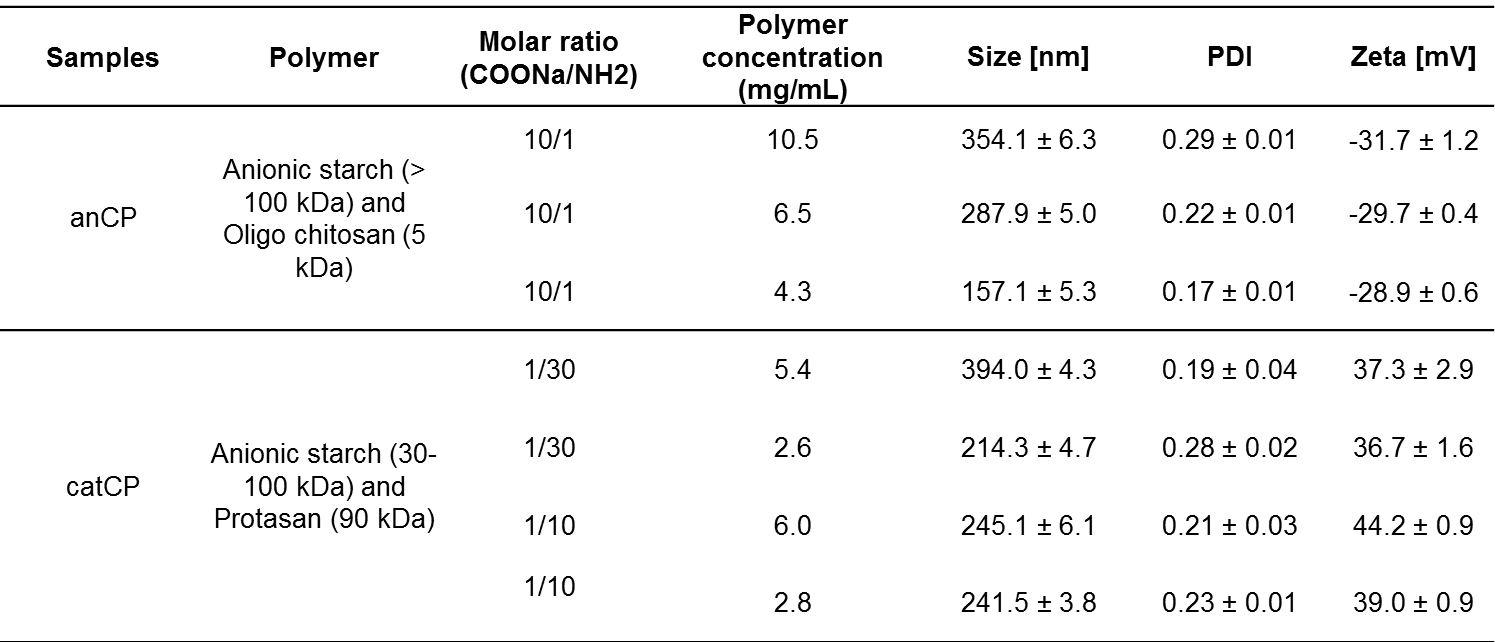


Table S3. Summary of anionic CP (anCP) and Protasan coated anCP (cCP) characteristics, in which anCP was produced with parameters, namely C/N ratio 10/1, and polymer concentration at 6.5 mg/mL. *N* > 3, n = 3, mean ± SD


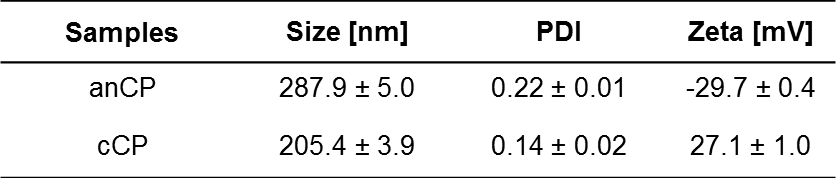

Figure S1. Physicochemical stability of starch-chitosan CP, in which anCP was produced with C/N ratio 10/1, and catCP was produced with C/N ratio 1/10, upon storage (4 ^o^C). The particles were diluted into MilliQ water at each time point for the measurement of size, PDI and ζ-potential. *N* = 3, n = 3, mean ± SD

Figure S2. Physicochemical stability of starch-chitosan anCP and cCP at different pH values ranging from 3.5 to 8.0, after 30 min and 1 h incubation. The initial pH- value of the samples was 5.5. *N* = 3, n = 3, mean ± SD

Table S4. Summary of tobramycin-loaded anCP characteristics achieved by variation of C/N ratio and polymer concentration. *N* > 3, n = 3, mean ± SD


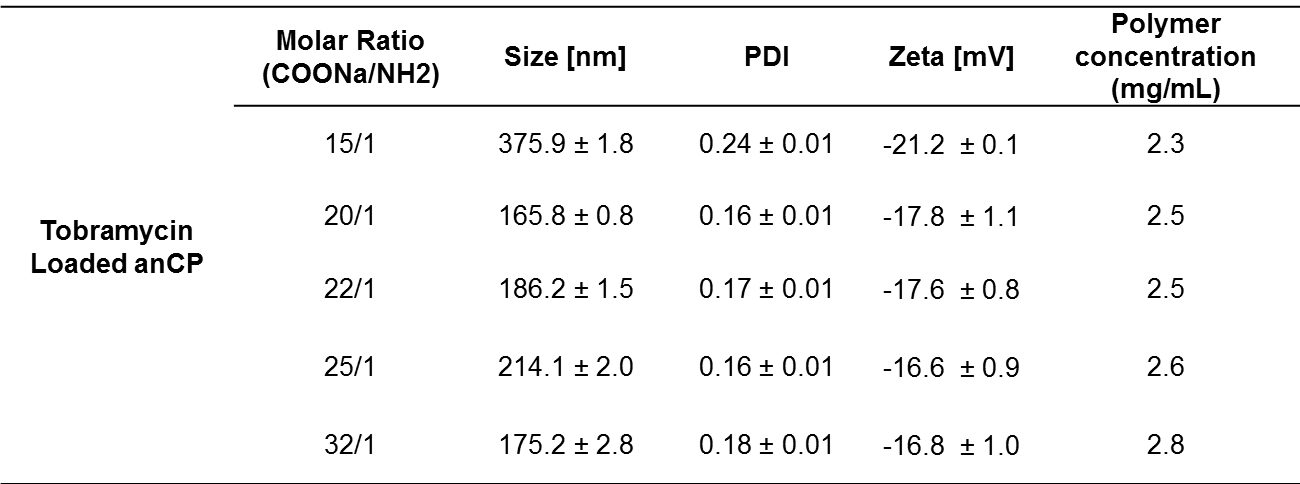


Table S5. Summary of colistin-loaded anCP characteristics resulting from variation of polymer concentration. *N* > 3, n = 3, mean ± SD


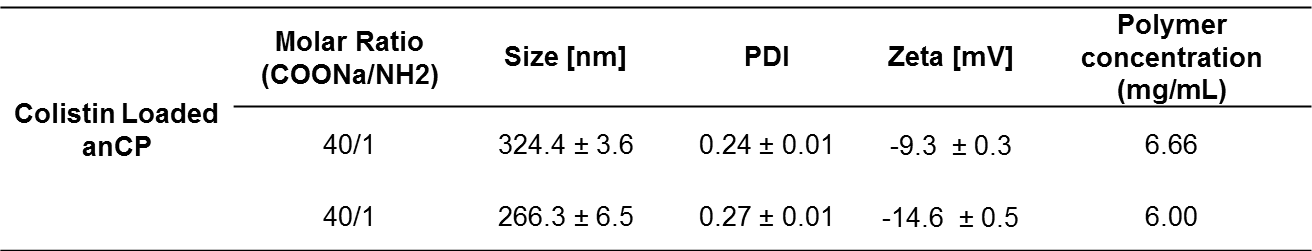


Table S6. Summary of drug loading quantification of tobramycin-loaded anCP. *N* > 3, n = 3, mean ± SD


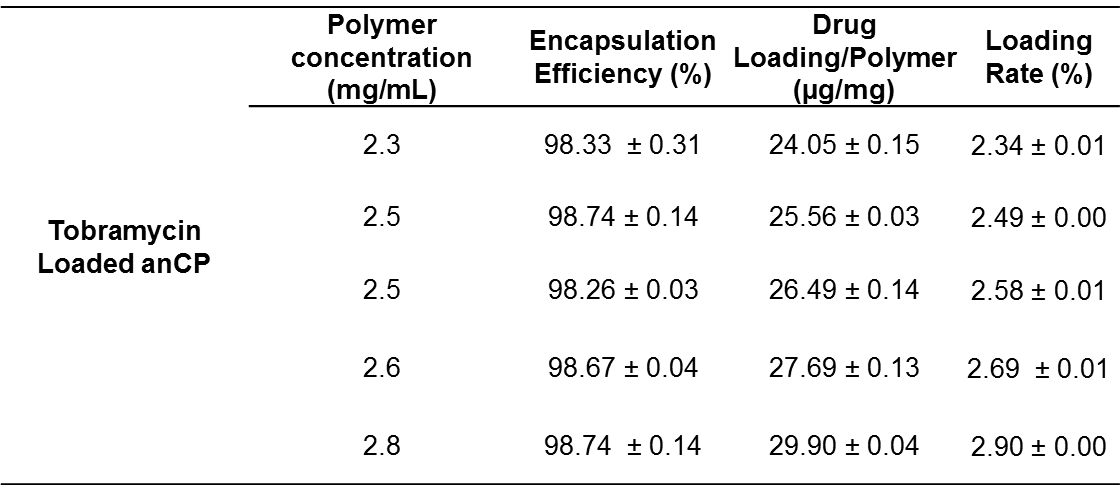


Table S7. Summary of drug loading quantification of colistin-loaded anCP. *N* > 3, n = 3, mean ± SD


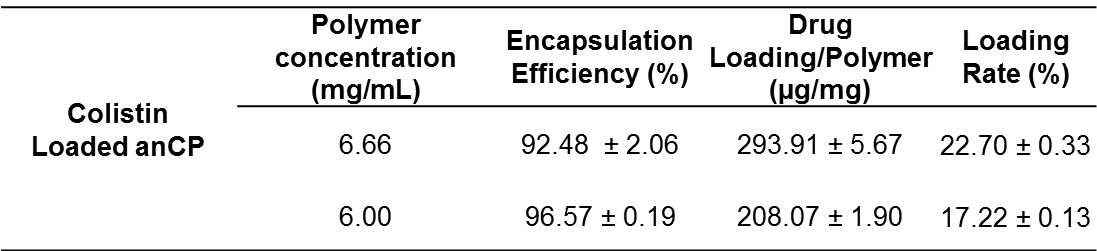


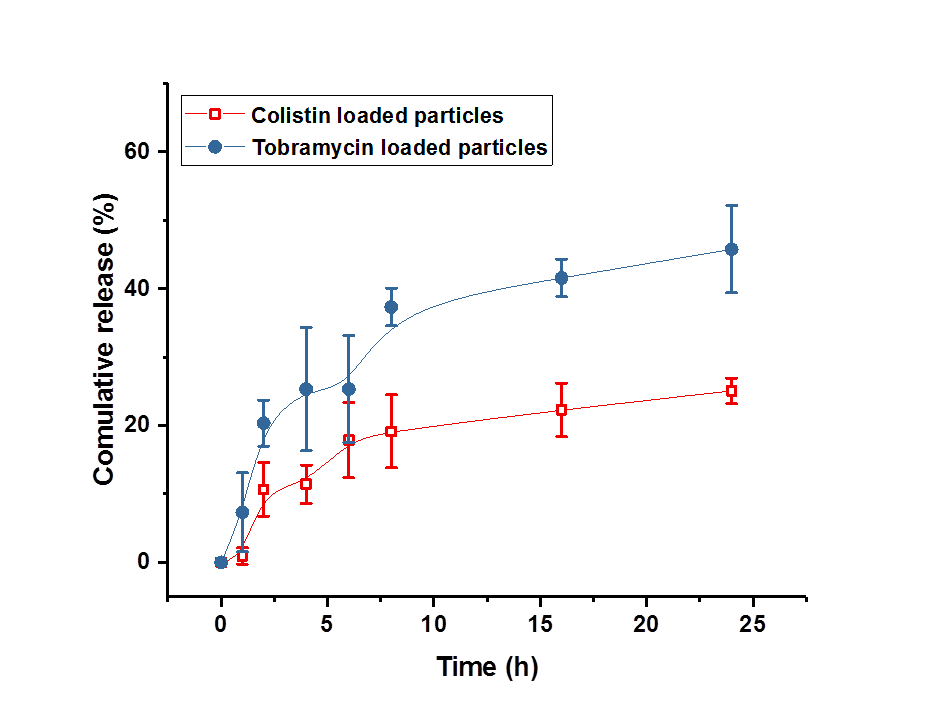


Figure S3. Cumulative release of tobramycin from tobramycin loaded anCP, and colistin from colistin loaded anCP performed in PBS at 37 °C. *N* = 3, n = 3, mean ± SD
